# Memories of paternal relations are associated with coping and defense mechanisms in breast cancer patients: an observational study

**DOI:** 10.1186/s40359-017-0206-z

**Published:** 2017-11-09

**Authors:** Chiara Renzi, Giada Perinel, Paola Arnaboldi, Sara Gandini, Valeria Vadilonga, Nicole Rotmensz, Angela Tagini, Florence Didier, Gabriella Pravettoni

**Affiliations:** 1 Applied Research Division for Cognitive and Psychological Science, European Institute of Oncology, Via Ripamonti 435, 20141 Milan, Italy; 2 Division of Epidemiology and Biostatistics, European Institute of Oncology, Via Ripamonti 435, 20141 Milan, Italy; 3 Department of Psychology, University of Milano-Bicocca, Piazza dell’Ateneo Nuovo 1, Milan, Italy; 4 Department of Oncology and Onco-Hematology, University of Milano, Via Festa del Perdono 7, Milan, Italy

**Keywords:** Coping, Defense mechanisms, Parental bonding, Breast cancer, Adjustment processes, Attachment theory

## Abstract

**Background:**

Breast cancer diagnosis and treatment represent stressful events that demand emotional adjustment, thus recruiting coping strategies and defense mechanisms. As parental relations were shown to influence emotion regulation patterns and adaptive processes in adulthood, the present study investigated whether they are specifically associated to coping and defense mechanisms in patients with breast cancer.

**Methods:**

One hundred and ten women hospitalized for breast cancer surgery were administered questionnaires assessing coping with cancer, defense mechanisms, and memories of parental bonding in childhood.

**Results:**

High levels of paternal overprotection were associated with less mature defenses, withdrawal and fantasy and less adaptive coping mechanisms, such as hopelessness/helplessness. Low levels of paternal care were associated with a greater use of repression. No association was found between maternal care, overprotection, coping and defense mechanisms. Immature defenses correlated positively with less adaptive coping styles, while mature defenses were positively associated to a fighting spirit and to fatalism, and inversely related to less adaptive coping styles.

**Conclusions:**

These data suggest that paternal relations in childhood are associated with emotional, cognitive, and behavioral regulation in adjusting to cancer immediately after surgery. Early experiences of bonding may constitute a relevant index for adaptation to cancer, indicating which patients are at risk and should be considered for psychological interventions.

## Background

Breast cancer is not only a cellular disease but also an event which requires adjustments in life-styles, body-image, and in family, couple and social dynamics [1, 2]. Women diagnosed with breast cancer often experience difficulties in this process. For instance, at pre-hospital admission, around 20% of breast cancer patients report intrusive thoughts and avoidance, while 70% report state anxiety [3]. Those with high symptom levels at diagnosis continue to experience them two years after diagnosis, and present difficulties in adjusting to the disease [3]. In this perspective, clinically significant symptom levels seem to persist in the long term, rather than representing a temporary condition. Importantly, this may lead to reduced treatment adherence and influence patient – health care professionals interactions (see e.g., [4]).

Adjustment to the disease, adherence to therapy, and interactions with healthcare professionals do not only depend on the characteristics of the disease but are associated to individual aspects of the patients [5]. Coping and defense mechanisms are two critical processes involved in adjustment to adverse situations such as the diagnosis of breast cancer. They were demonstrated to be inter-related, in the sense that even if they rely on different theoretical backgrounds and describe distinct psychological constructs, both aim at dealing with negative emotions and at restoring homeostasis. Criteria that differentiate between defense and coping processes include the conscious/unconscious status and the intentional/nonintentional nature of the processes. Criteria based on the dispositional or situational status of the process, and on the conceptualization of the processes as hierarchical, are demonstrated to be more a matter of overlap than of difference [6]. For instance, while the dispositional aspect of defense mechanisms is often theoretically emphasized in contrast to coping intended as strategies specific to a particular event, research indicated that both coping and defense mechanisms are influenced by personality traits as well as by the context [6 for a review].

When facing the diagnosis of breast cancer, women employ more or less adaptive coping strategies which depend both on the dispositional traits as well as on situational traits such as the phase of the disease. Dysfunctional coping mechanisms are related to less adaptive illness behaviors and psychological distress in cancer patients [7, 8]. For instance, the rigid use of avoidance may compromise active engagement of patients’ in the illness clinical pathway [3], and threaten the use of important resources such as social support [9]. However, by means of this strategy, the patient may also minimize stress by avoiding, for instance, those social interactions which may require talking about the oncological disease [10].

The use of defense mechanisms may be triggered in the attempt to protect the individual from feelings or needs which could expose the individual to excessive affective activation [6, 11]. In general, the use of defense mechanisms is considered a function of the human mind and partly dispositional, however the flexibility in their use, their effectiveness, the hierarchical level of the defense (whether immature of mature, see below), and the situational characteristics may provide indications of pathological functioning [6]. Effective use of defenses in medically hospitalized patients was found associated with better psychological adjustment, while ineffectiveness in the mechanism was related to psychological distress [12]. Lower level or “immature” defenses (e.g., splitting or denial) are positively correlated with measures of psychopathology, while higher level or “mature” defenses are positively correlated with better psychological adjustment [13, 14]. Denial may result in delay for undergoing breast biopsy in the suspect of breast cancer, while its use is associated with reduced distress in women with a diagnosis of breast cancer [12]. Denial would thus protect the individual from experiencing an affect associated with the idea of having breast cancer, but depending on when and how the defense is triggered, it may result as adaptive or not.

However, since coping and defense mechanisms to cancer can be evaluated only at the time of their enactment, it is important to consider factors that may contribute to emotional, cognitive, and behavioral programming and regulation, and may thus provide information on the ability of the individual to adjust to stressful situations. In fact, coping and defense mechanisms are not only related to the characteristics of the event itself, but also depend on patterns acquired through relevant affective relationships, which modulate the subjective perception of an event as stressful and the development of adaptive processes.

According to the adult attachment theory, the possibility to receive care and protection when in need during childhood, while allowing for a safe exploration of the environment in other moments [15] is a premise to develop a condition of equilibrium with a good regulation and modulation of emotional experiences in adulthood. Under different circumstances, individuals may develop poorly regulated affection, or rigidly organized affective patterns, or present dysregulated and inconsistent affective responses [15]. Therefore, the way a potential stressor is processed and the undertaken responses to manage it are likely to be related to the subjective biographical experience of first interactions [11, 16].

Children who experienced adequate parental relations are more likely to acquire the ability to master negative emotions independently [17, 18], and to cope with adverse life situations in adulthood by using more functional cognitive and affective strategies [19–23]. On the other hand, inadequate parental relations may lead to a more frequent activation of immature defense mechanisms.

Importantly, early parental relations (including attachment patterns) can influence interactions with healthcare professionals in breast cancer patients [24–29], thus suggesting that they may modulate more in general adjustment processes after the diagnosis. Breast cancer patients’ attachment model but not surgeon’s identity was modestly but significantly associated with the perceived alliance with breast cancer surgeons [26]. Similarly, in a sample of breast cancer patients attending a follow-up clinic, those with positive models of self, perceived more support from nurses [27].

In the present exploratory study, we assessed coping styles, defense mechanisms and recollected parental caregiving style in women at their first breast cancer diagnosis in the early post-operative phase (1–7 days after quadrantectomy or mastectomy as a first therapeutic approach). It was hypothesized that the quality of parental relations as recollected would be associated with the adaptiveness of coping strategies and defenses in this phase. To our knowledge, this is the first study investigating the association between the recollection of early parental caregiving and adjustment processes following breast cancer surgery.

## Method

### Participants

Inpatients were recruited between September 2011 and June 2012 during hospitalization in the Breast Cancer Unit of the European Institute on Oncology in Milan, Italy. All women were diagnosed with primary breast cancer and had not received the histopathological results at the time of assessment. Inclusion criteria were: first diagnosis of breast cancer, absence of major psychiatric diseases or severe neurological events that could interfere with test completion. Exclusion criteria were: neo-adjuvant therapy. A total of one hundred fifty-four women were approached. Five women refused to participate due to lack of time, fatigue, or post-surgical pain. Fourty-three women agreed to participate and gave their informed consent but had incomplete assessments or did not return the questionnaires. A total of 110 women participated in the study after written informed consent was obtained (mean age = 50, range 29–65) and had complete assessesments. Patients underwent quadrantectomy (*N* = 90) or mastectomy (*N* = 20) as a first therapeutic approach.

Patients with histologically confirmed diagnosis of breast cancer were identified via two databases: the Institutional Breast Cancer Database and the Tumor Registry of the European Institute of Oncology (IEO). The study was approved by the IEO Institutional Review Board.

The authors confirm that all procedures contributing to this work comply with the ethical standards of the relevant National and institutional committees on human experimentation and with the Helsinki Declaration of 1975, as revised in 2008.

Demographic data, clinical data and life-style variables were recorded in a case record form. The characteristics of the sample are shown in Table 1.

**Table 1:** Socio-demographic characteristics and tumor features of patients included

		Number	Percent
Age median (Q1-Q3)		50 (43–-55)	
Marital status	Married/co-habitant	88	80%
	other	22	20%
Educational level	Elementary	33	30%
	Middle school	29	26%
	High school	48	44%
	MSC/PHD	29	26%
Socio-Economic Status	High	8	7%
	Middle	74	67%
	Low	27	25%
Parity	0	33	30%
	1 child	33	30%
	>1 children	44	40%
T stage	0	1	1%
	I	67	61%
	II-IV	42	38%
Lymph-node involvement	No	53	48%
	Yes	57	52%
Mastectomy	No	90	82%
	Yes	20	18%

Socio-Economic Status: “Low” corresponds to housewife or unemployed; “Middle” corresponds to clerk, employee, worker, laborer, teacher and retired; “High” corresponds to: executive, freelance, medical doctor, architect, engineer, etc.

### Instruments

#### Recollection of parental relations - parental bonding instrument (PBI)

The Italian version of the PBI was used to evaluate the quality of primary relations as recollected in adulthood [30]. The instrument is a self-report composed of 25 items measuring two distinct dimensions: parental care and overprotection. The individual is asked to evaluate the degree of accord with the sentences presented with respect to her subjective experience of the first 16 years of life, with maternal and paternal figures on a 4-point Likert-scale. Maternal and paternal bonding are rated on two separate questionnaires. Cut-off scores of the questionnaire (for mothers, a care score of 27.0 and a protection score of 13.5; for fathers, a care score of 24.0 and a protection score of 12.5) indicate whether parents were high or low on the dimensions of care and overprotection.

The PBI does not directly measure the state of mind with respect to attachment relations. In fact, being a self-report instrument, it represents the perceived or remembered style of parental caregiving rather than the actual quality of attachment. The PBI showed convergent validity with the Adult Attachment Interview for optimal relations and secure attachment [31]. In this sense, optimal parental caregiving is a ‘correlate’ of secure attachment relations. Amongst the self-administered questionnaires assessing the dimensions of attachment, the PBI is indicated as one of the most solid [32], with good internal consistency and test-retest reliability [33], satisfactory construct and convergent validity [34], and stability over a 20 years interval [35]. Furthermore, it is independent of mood effects [34].

#### Coping - mini-mental adjustment to cancer scale (mini-MAC)

The Italian version of the Mini-MAC [36] is a 29 items instrument which measures cognitive and behavioral responses to cancer on a 4-points Likert scale. Items can be grouped on five categories representing different coping styles. The H*elplessness/Hopelessness* category represents high levels of anxiety and depression, absence of cognitive strategies that may allow acceptance of the diagnosis, use of unaimed behavioral responses. *Anxious preoccupation* is defined by constant worry about the disease, and feelings of anxiety, fear and apprehension. *Fighting spirit* is characterized by moderate levels of anxiety and depression, use of confrontation (positive thinking), palliative (reducing the impact of the diagnosis), and behavioral responses. *Avoidance* reflects the absence of anxiety and depression, and the predominant use of cognitive strategies. *Fatalism/Stoic acceptance* is characterized by low levels of anxiety and depression, loss of internal control, and fatalistic attitudes. Items assigned to each coping styles are summed to obtain a total score representing the degree of use of each coping style.

#### Defense mechanisms - response to evaluation measure – 71 (REM-71)

The Italian version of the REM-71 [37] is a self-report questionnaire consisting of 71 items to evaluate defensive strategies. Defenses are divided in two categories: Factor 1 corresponds to unadaptive or immature defenses, while Factor 2 corresponds to more adaptive e flexible ones. Defenses here are defined as reactions of which the individual is unaware, reflecting both innate traits and learned coping mechanisms which are not necessarily pathological and may exclude information from awareness [38]. A total of 21 defense mechanisms (each composed of three or four items) are evaluated on a 9-point Likert scale. Scores assigned to items, referring to each defense mechanism are summed to form a defense mechanism score, and can be further calculated to obtain Factor 1 (immature) and Factor 2 (mature) scores. Factor1 includes 14 defenses namely acting out, conversion, displacement, dissociation, fantasy, omnipotence, passive aggression, projection, repression, somatization, splitting, sublimation, undoing, withdrawal. Factor2 includes 7 defenses namely altruism, isolation of affect, humor, idealization, intellectualization, reaction formation, suppression. Cronbach’s alpha for single defenses ranges between 0.36 e 0.85 (mean value of the coefficient = 0.56), while it corresponds to 0.84 for Factor1 and 0.68 for Factor2 [37]. Even if the alphas for some subscales of the REM-71 reported in Prunas et al. [34] are low, they were used because the subscales may be more informative than the two broad factors. An evaluation of reliability of subscales in this population was performed.

### Procedures

A clinical psychologist approached patients on day 1 or 2 after surgery in the ward. After careful explanation of the study procedures and informed consent procedures, an appointment was scheduled. In the majority of cases, tests were completed during hospitalization. When this was not possible, the appointment was scheduled on the same day of surgical follow-up (within a week from discharge).

### Statistical methods

Descriptive statistics (median and interquartile ranges - IQR) and frequencies were used to describe patients’ socio-demographic features and relevant clinical variables.

Spearman correlations coefficient and *P*-values for the correlation between coping and defenses are presented.

Cronbach’s Coefficient Alphas of subscales of the REM-71 were recalculated for the present study.

Care and Overprotection dimensions of parental relations were categorized in ‘high’ and ‘low’ considering the cut-off scores of care and overprotection from Parker and collaborators [33]. Associations between Coping Styles and Defense Mechanisms with Parental Style (Care and Overprotection), possible confounding factors (age, BMI, menopausal status, family history, parity, education, marital status), types of treatments (mastectomy or quadrantectomy) and other cancer prognostic factors were assessed by univariate analyses (Wilcoxon-rank tests and Spearman correlations coefficient) in order to identify variables to be included in the multivariate ANCOVA models.

*P*-values from multivariate ANCOVA models, indicating Defense Mechanisms and Coping Styles associated with Care and Overprotection, adjusted for significant confounding factors and other cancer prognostic factors, are presented.

Residuals from full model were checked to verify normal distribution.

Two-sided P-values were used in the analyses. The criterion for statistical significance was set at 5%. Data were analyzed using the SAS System Software for Windows, release 9.2. (SAS Institute, Cary, NC, USA).

## Results

### Descriptives

Table 1 indicates socio-demographic features of the 110 patients with tumor characteristics and type of surgery. 20% of patients lived alone, 30% had no children, 30% obtained an elementary school diploma and 25% were classified as “low socio-economic status” based on their jobs. Half of the patients (52%) had lymph-node involvement, 18% of them had a mastectomy.

Median scores and IQR ranges for parental relations, coping styles and defense mechanisms are shown in Table 2. Mother’s and father’s care median equaled the cut-off score (Mother: median = 27, IQR 17–31; Father: median = 25, IQR 18–32). Median overprotection scores were higher than the cut-off score (Mother: median = 14, IQR 10–31; Father: median = 15, IQR 9–20). Anxious preoccupation was the coping style with the highest scores (median = 19; IQR 15–22), followed by fighting spirit (median = 16; IQR 14–17), hopelessness/helplessness (median = 14; IQR 11–18), avoidance (median = 11; IQR 9–13), and fatalism (median = 11; IQR 9–12).

**Table 2:** Median value and interquartile range of coping, parental relations and defenses

Variables	Median	Lower Quartile	Upper Quartile
Coping			
Anxious preoccupation	19	15	22
Avoidance	11	9	13
Fatalism	11	9	12
Fighting spirit	16	14	17
Hopelessness/Helplessness	14	11	18
Defenses			
**Factor1**	**4.05**	**3.40**	**4.69**
Acting	3.67	2.33	4.67
Conversion	1.00	1.00	1.67
Displacement	2.67	2.00	4.00
Dissociation	4.00	2.67	5.33
Fantasy	3.00	1.33	4.67
Omnipotence	4.67	3.33	5.67
Passive aggression	4.00	3.33	5.67
Projection	2.33	1.33	3.00
Repression	3.33	1.67	4.67
Somatization	4.33	2.67	5.67
Splitting	6.33	5.33	7.67
Sublimation	5.33	4.33	6.67
Undoing	4.33	3.33	6.00
**Factor2**	**5.64**	**4.87**	**6.14**
Altruism	8.00	7.25	8.75
Denial	4.83	3.67	6.00
Humor	5.13	3.50	6.50
Idealization	6.50	5.33	8.00
Intellectual	5.25	4.25	6.50
Reaction formation	4.33	3.00	5.67
Suppression	5.33	3.67	7.00
Withdrawal	6.33	4.33	7.67
Parental relations			
Care – Father	25	18	32
Care – Mother	27	17	31
Overprotection – Father	15	9	20
Overprotection – Mother	14	10	21

Median values of Factor 2 (mature) defenses were higher than Factor 1 (Factor 1: median = 4.05, IQR 3.40–4.69; Factor 2: median = 5.64, IQR 4.87–6.14). Altruism was the most used defense in the sample (median = 8.0, IQR 7.25–8.75), followed by idealization (median = 6.5, IQR 5.33–8), splitting (median = 6.33, IQR 5.33–7.67) and withdrawal (median = 6.33, IQR 4.33–7.67).

### Relation between coping and defense mechanisms

In order to explore the relation between defenses and coping strategies, a correlation analysis using Spearman’s coefficient was run (see Table 3). Results showed that higher Factor1 scores significantly correlated with the adoption of helplessness/hopelessness and avoidance coping styles (*ρ*_*s*_ = 0.21, *p* = 0.027; *ρ*_*s*_ = 0.33, *p* < 0.001 respectively). Factor2 scores were inversely correlated to the use of helplessness/hopelessness and anxious-preoccupation coping styles *(ρ*_*s*_ = −0.32, *p* < 0.001; ρ_s_ = −0.36, *p* < 0.001 respectively), while they were positively correlated to fatalism and fighting spirit (*ρ*_*s*_ = 0.23, *p* = 0.014; *ρ*_*s*_ = 0.34, *p* < 0.001 respectively). The pattern was maintained when considering only patients who underwent quadrantectomy.

**Table 3:** Spearman correlation coefficients and P-values for coping and defense factors

		Coping					
		Hopelessness	Anxious preoccupation	Fatalism	Fighting spirit	Avoidance	
Defenses	Factor1	0.21	0.15	0.18	0.05	0.33	Spearman’s ρ
		**0.03**	0.11	0.06	0.63	**<0.001**	*p-values*
	Factor2	−-0.32	−-0.36	0.23	0.34	0.09	Spearman’s ρ
		**<0.001**	**<0.001**	**0.01**	**<0.001**	0.33	*p-values*

Significant *p*-values are indicated in bold

### Relation between recollected parental bonding and adjustment processes

**Fig. 1:**
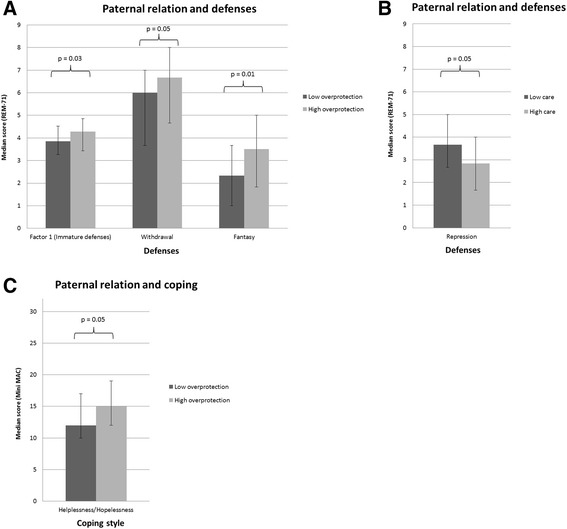
Panel **a**) Bars depict median values of all Factor 1 defenses, and of Withdrawal and Fantasy defense by Paternal Overprotection. Dark grey bars represent the values for patients with low paternal overprotection, while light grey bars represent the values for patients with high paternal overprotection. Panel **b**) Bars depict median values of Repression defense by Paternal Care. Dark grey bars represent the values for patients with low paternal care, while light grey bars represent the values for patients with high paternal care. Panel **c**) Bars depict median values of Hopelessness/Helplessness coping style by Paternal Overprotection. Dark grey bars represent the values for patients with low paternal overprotection, while light grey bars represent the values for patients with high paternal overprotection

Table 4 and Fig. 1 present median values and IQR ranges of coping styles and defenses, by type of relation with the father (care and overprotection) categorized in high and low based on the cut-off value from Parker and colleagues [33].

**Table 4:** Median value and range interquartile of coping and defenses by type of attachment dimension with the father

	cut off^a^	Response Variable	Number	Median	Q1	Q3	*p*-values^b^
Paternal overprotection	≤12.5	Factor1	45	3.86	3.26	4.52	0.03
	>12.5		64	4.27	3.43	4.86	
	≤12.5	Withdrawal		6.00	3.67	7.00	0.01
	>12.5			6.67	4.67	8.00	
	≤12.5	Fantasy		2.33	1.00	3.67	0.05
	>12.5	Fantasy		3.50	1.83	5.00	
	≤12.5	Hopelessness		12	10	17	0.05
	>12.5	Hopelessness		15	12	19	
Paternal care	≤24	Repression	53	3.67	2.67	5.00	0.05
	>24		56	2.83	1.67	4.00	

^a^from Parker et al. [33]

^b^Multivariate ANCOVA models with Paternal overprotection and Paternal care as explanatory variables, adjusted for age and mastectomy

*P*-values are obtained from the multivariate ANCOVA model assessing the association between coping styles and defenses, and father care or overprotection, adjusting for age and type of surgery as confounding variables. Patients who reported high levels of overprotection in the relation with their father had significantly higher scores on Factor1 defenses on the REM-71 measure. Cronbach’s coefficient of Factor1 from the present sample is 0.79. Similar results were found considering two specific defenses: fantasy and withdrawal. Cronbach’s coefficient of these subscales indicate that they are reliable (fantasy: α = 0.60; withdrawal: α = 0.80). These patients also exhibited higher levels of helplessness/hopelessness coping strategies on the Mini-MAC measure. No significant association was found for other coping strategies. On the contrary, those women who reported high levels of care in the attachment relation with the father also had lower scores on the repression scale (included in Factor1 defenses). No significant association was found for the reported attachment relation with the mother.

## Discussion

The results of the present study indicate that recollected characteristics of the relationship with the paternal figure are significantly associated with defense mechanisms and coping strategies. Furthermore, these adaptative mechanisms are related to each other, shortly after the diagnosis of breast cancer, i.e. in the surgical phase of treatment. In particular, elevated overprotection is associated to the reported use of immature defenses and helplessness/hopelessness coping with cancer, while low care was associated to a higher incidence of repressive defenses.

These data provide support to the hypothesis that early parental relationships may be related to psychological adjustment in breast cancer. Coherently, insecure attachment (more frequently found in conditions of non-optimal parenting style) was related to a less flexible use of coping strategies in a group of patients with chronic disease, including a sample of women with breast cancer [39]. Patients with secure attachment (more frequently found in conditions of optimal parenting style) employed social resources more often [40] as well as other strategies, classified as adaptive [41]. On the contrary, women with a history of anxious-ambivalent attachment had negative emotion strategies to a greater degree, and reported higher levels of helplessness/hopelessness and anxious preoccupation coping strategies [39, 42].

Despite research (e.g., [39]) reporting that the maternal relationship may modulate psychological adjustment to adverse life events, no association with the maternal relationship was found. A possible hypothesis that can sustain this result is that patients’ memories could be influenced by the affective mental status that characterizes breast cancer diagnosis: the area of the breast is strictly connected with femininity and maternal aspects, and patients could have been frail and sensitive in recollecting their maternal bond during their hospitalization for surgery. Another explanation may be related to the variability of data and the lack of statistical power with the present sample.

The present data pointed to the role of the paternal relationship as an important predictor of adaptive adjustment processes. These evidences are in accordance with experimental, clinical and epidemiological studies that provide evidence of phenotypic and epigenetic effects mediated via the paternal line [43, 44]. It has been hypothesized that the importance of the paternal relationships resides in learning how to cope with environmental challenges. In fact, interaction with fathers has been described as involving surprise and encouragement in challenging scenarios during which children learn to experience risks and courage [45]. Fathers’ sensitivity in challenging their two years old toddlers during exploration was predictive of greater security of coping with feelings of sadness, anger or fear, positively correlated to reported active coping styles, and negatively correlated to problem avoidance in adolescent daughters at an older age. On the contrary, more frequent reprimands and greater intrusiveness during play were positively correlated to greater problem avoidance, and negatively correlated to active coping styles [46].

The present study’s results are in line with such evidence, showing that a recollected greater paternal control is associated with the use of a helplessness/hopelessness strategy, which is characterized by a pessimistic and passive attitude [36]. This coping style is considered dysfunctional during the first phase of the disease since surrendering to cancer may, in fact, become an obstacle to treatment adherence and to the patient-clinician relation [4]. As a consequence, the patient’s quality of life during the disease may be reduced. Critically, the use of a hopelessness/helplessness coping style in cancer patients positively correlates with the presence of depression and anxiety while the opposite is found for fighting spirit coping [47, 48]. In turn, helplessness and depression are associated with shorter cancer survival (e.g., [49]).

The data of the present study also indicate that high levels of control and low levels of care experienced with fathers led to a greater control on emotional reactions in adulthood, thus recruiting more rigid and controlling defensive styles. In fact, the defenses found associated to paternal styles are characterized by a component of negation and avoidance of reality that, in the case of breast cancer patients, may exclude the cognitive and emotional impact of the disease. In particular, low levels of paternal care were associated with the use of repression as a defense mechanism. In this case, disturbing thoughts, wishes or experiences are expelled from conscious awareness. On the other hand, high levels of overprotection were linked to withdrawal and fantasy as defenses. The former reflects a state of apathy, characterized by emotional indifference, and a reduction of social contacts and activities that leave individuals passive to events and with respect to caregivers. Fantasy refers to daydreaming as a substitute for human relationships, effective actions, or problem solving. Daydreaming and engagement in self-comforting fantasies was previously found to be associated with a negative prognosis in breast cancer patients [50].

High paternal overprotection and insecure attachment are related to the development of psychological disorders such as depression [51–53]. Immature defenses and depression predict shorter survival in late-stage cancer [54]. Notably, while defensive style is predictive of 5 years survival 8 months after assessment, depression was found to be predictive only 30 months after the assessment [54].

Defense mechanisms and coping strategies are linked [55, 56], and this seems to be the case also in our sample. In fact, a significant positive correlation was found between the use of Factor1 defenses and the adoption of helplessness/hopelessness and avoidance coping styles. In addition, a significant association between Factor2 defenses and fighting spirit was found. Factor 2 was also positively correlated to fatalism, and negatively correlated to helplessness/hopelessness and anxious-preoccupation coping styles. Similar to previous studies (e.g., [57]), these results point to a correspondence between mature defenses and adaptive coping strategies, and between immature defenses and dysfunctional coping styles in breast cancer [55].

It may be hypothesized that the type of surgery, and in particular its impact on the body image (which is dramatically higher for mastectomy), could play a role in the perceived stressfulness of the event and thus on the type of adaptive processes activated. This factor was not found to be significant in the analysis of confounds, nevertheless the results were corrected for type of surgery since it is possible that the reduced number of patients who underwent mastectomy was not sufficient to guarantee adequate statistical power.

From a clinical perspective, our results suggest that recollected significant relationships play a role in the modulation of adult responses to stressful events. In fact, insecure parental relations in childhood are often linked to dysregulation of emotions, and to a reduced ability to express needs and to mobilize internal resources in adulthood. Importantly, these aspects may be reflected in the interactions and levels of cooperation with clinical staff in a potentially stressful situation such as breast cancer treatment [24, 28], in which the activation of the attachment motivational system may be more likely. Breast cancer patients with a positive attachment model are more likely to report receiving full support from nurses [27] and to develop an alliance with breast cancer surgeons compared to women with less positive models [26].

The limits of the present study lay in its observational nature and in the relatively small sample, which does not allow to draw definitive conclusions on the direction of the associations that were found. For instance, rigid defensive styles and the enactment of dysfunctional coping styles may have influenced the reports of caregiving styles as well as non-optimal parenting may lead to the use of immature defenses and anxious or helpless/hopeless coping styles. This is also connected to the use of self-report measures that, in this case, were chosen for their lower intrusiveness and their easier implementation in the schedules and practices of the hospital setting. Starting from these result, future studies may use a different study design and benefit from the use of different scales that do not implicate self-report, such as the Adult Attachment Interview [58]. Yet, the use of a homogeneous sample (all women at their first diagnosis of breast cancer, who underwent surgery as the first therapeutic approach) provides a solid picture of the adjustment mechanisms that partially overcomes the bias intrinsic to the self-report, the phase being the same for all patients. Further research may also consider the temporal development of adjustment mechanisms in light of parental relations and internal working models.

## Conclusions

The association found between coping styles, defense mechanisms and early parental relations suggests that the evaluation of relational history in the psycho-oncological context may provide an additional prognostic index of adjustment abilities, thus indicating which individuals are at risk and may need support after diagnosis.

Previous studies demonstrated that psychological treatment for cancer patients determines an increase of active coping [59], and decreases mortality and recurrence rates at a 10 year follow-up [60]. Importantly, changes in active coping did predict clinical outcomes, and may thus mediate the relation between changes in immunological parameters and prognosis [59, 60].

In this view, psycho-oncological assessments should not overlook the investigation of developmental history, and in particular relations with caregivers, to implement personalized care reflecting the single patients’ characteristics and needs. These evidences support the development of personalized medicine approach [5, 61] that takes into consideration the subjective characteristics of patients including personality predisposition to a particular kind of patient-health care professional relationship.
